# Inverted Oscillator Quantum States in the Probability Representation

**DOI:** 10.3390/e25020217

**Published:** 2023-01-22

**Authors:** Olga V. Man’ko, Vladimir I. Man’ko

**Affiliations:** 1 Lebedev Physical Institute, Russian Academy of Sciences, Leninskii Prospect 53, Moscow 119991, Russia; 2 Russian Quantum Center, Skolkovo, Moscow 143025, Russia

**Keywords:** probability distributions, quantizer operator, dequantizer operator

## Abstract

The quantizer–dequantizer formalism is used to construct the probability representation of quantum system states. Comparison with the probability representation of classical system states is discussed. Examples of probability distributions describing the system of parametric oscillators and inverted oscillators are presented.

## 1. Introduction

In classical mechanics, the states of classical particles are described by the two numbers—position *q* and momentum *p*. The time evolution of the states is associated with the trajectory q(t) and $p\left(t\right)=m\dot{q}\left(t\right)$. The evolution is described by the Newtonian law $m\ddot{q}\left(t\right)=F$, where force *F* is determined by the potential energy V(q) term in the Hamiltonian $H=p^{2}/2m+V\left(q\right)$. In the case of classical statistical mechanics, the particle states are described by the probability density function f(q,p,t) of the two random position and momentum and the time *t*. The probability density satisfies the evolution equation

(1) $$\frac{\partial f(q,p,t)}{\partial t}+\frac{\partial f(q,p,t)}{\partial q}\frac{p}{m}-\frac{\partial f(q,p,t)}{\partial p}\left(\frac{\partial V}{\partial q}\right)=0.$$

For quantum systems, the conventional description of quantum states, called pure states, is formulated using the concept of the complex wave function ψ(q,t)=|ψ(q,t)|exp(iϕ(q,t)). The physical meaning of the wave function modulus squared ${\left|\psi \left(q,t\right)\right|}^{2}dq=w\left(q,t\right)dq$ is the probability density at time *t* in the interval dq around position *q*. Due to this, the wave function is normalized, i.e., ${\int |\psi \left(q,p\right)|}^{2}dq=1$. The analogous concept of probability density for a classical particle is expressed in terms of the function f(q,p,t), namely $w_{cl}\left(q,t\right)=\int f\left(q,p,t\right)dp$. The position and momentum in quantum mechanics are the operators $\hat{q}$ and $\hat{p}$ acting on the wave function in position representation as $\hat{q}\psi \left(q,t\right)=q\psi \left(q,t\right)$. $\hat{p}\psi \left(q,t\right)=-i\hbar \frac{\partial \psi (q,t)}{\partial q}$, where *ℏ* is the Planck constant and the position and momentum operators do not commute, i.e., $\left[\hat{q},\hat{p}\right]=\hat{q}\hat{p}-\hat{p}\hat{q}=i\hbar \hat{1}.$ This property means that the probability density of position and momentum does not exist since there exists an uncertainty relation of position and momentum ${\left(\delta q\right)}^{2}{\left(\delta p\right)}^{2}\geq \hbar^{2}/4$ [1], which does not exist in classical mechanics. The evolution of the quantum state is described by the Schrödinger equation [2]

(2) $$i\hbar \frac{\partial \psi (q,t)}{\partial t}=\left(\frac{{\hat{p}}^{2}}{2m}+\hat{V}\left(\hat{q}\right)\right)\psi \left(q,t\right).$$

The particle statistical properties (mean values 〈q〉,〈p〉) and dispersions ($\langle q^{2}\rangle -{\langle q\rangle }^{2},$$\langle p^{2}\rangle -{\langle p\rangle }^{2}$) are expressed in terms of the wave function ψ(q,t). For example,

(3) $$\langle q\rangle ={\int q|\psi \left(q,t\right)|}^{2}dq,\;\langle q^{2}\rangle =\int q^{2}{\left|\psi \left(q,t\right)\right|}^{2}dq.$$

For the classical particle, we have the means of position and momentum

(4) $${\langle q\rangle }_{cl}=\int qf\left(q,p,t\right)dqdp,\;{\langle p\rangle }_{cl}=\int pf\left(q,p,t\right)dqdp.$$

An important property of quantum states is the superposition principle of any states with wave functions $\psi_{1}\left(q,t\right)$ and $\psi_{2}\left(q,t\right)$. According to this principle, the function $\psi \left(q,t\right)=c_{1}\psi_{1}\left(q,t\right)+c_{2}\psi_{2}\left(q,t\right)$ corresponds to a real state which exists. Here, the numbers $c_{1}$ and $c_{2}$ are complex numbers. To describe the quantum states of a particle in an environment of mixed states, for example, at the temperature *T*, the concept of the wave function is not sufficient and the notion of the density matrix $\rho \left(x,x^{\prime },t\right)=\sum_{k}\phi_{k}\psi_{k}\left(x,t\right)\psi_{k}\left(x^{\prime },t\right)$, which is a convex sum of terms expressed using wave functions, was introduced [3,4]. The numbers $\phi_{k}$ in the convex sum are probabilities satisfying the normalization condition $\sum_{k}\phi_{k}=1$. Thus, the notion of the state of the particle in classical mechanics (and classical statistical mechanics) is dramatically different from the notion of state in quantum mechanics. Moreover, the notion of observables, such as position and momentum, in quantum mechanics uses the position and momentum operators ($\hat{q}$ and $\hat{p}$) acting in the Hilbert space of state vectors |ψ〉 [5]. Due to these differences in classical and quantum mechanics formulations, from the very beginning of the introduction of quantum mechanics, attempts to find a formulation closer to the classical one were undertaken. The idea was to introduce a description of state similar to introducing the notion of the probability density f(q,p). Quasiprobability distributions were introduced by Wigner [6] and other versions of quasiprobability distributions were described by Husimi [7]. Glauber [8] and Sudarshan [9] introduced another version of the quasiprobability function, describing the particle states. All these functions are functions of two variables *q* and *p*, but they cannot be analogs of position and momentum, as in classical mechanics, because the Heisenberg uncertainty relation forbids the existence of such probability distributions. The problem was solved [10] when the probability distribution function of one random position *X* was found (see also [11,12,13]). This probability distribution contains all the information about the quantum state of a particle and is related to the density matrix and all known quasidistrubition functions by invertible integral transforms. The aim of the article is to present the construction of such probability representations of quantum states and to consider some examples, such as parametric and inverted oscillator states in this new probability representation of quantum mechanics. The inverted oscillator was recently discussed in [14,15] in relation to cosmological problems. Some of the inverted oscillator problems were also considered in [16,17,18,19].

The paper is organized as follows. In Section 2, the formalism of quantizer–dequantizer operators [20] is reviewed. In Section 3, the Hamiltonian and integrals of linear motion in the position and momentum of oscillators are discussed. In Section 4, the states of the inverted oscillator and the Shannon entropy are considered. In Section 5, the evolution of the inverted oscillator states is considered. The conclusions and future directions are presented in Section 6.

## 2. Radon Transform and Quantizer–Dequantizer Operators

In order to clarify the possibility of introducing the probability representation of quantum states, let us first consider the analog of introducing the specific probability distribution in classical statistical mechanics. We consider the Radon transform [21] of the probability density of two random variables f(q,p) of the form

(5) $$w_{cl}\left(X|\mu ,\nu \right)=\int f\left(q,p\right)\delta \left(X-\mu q-\nu p\right)dqdp.$$

The inverse Radon transform reads

(6) $$f\left(q,p\right)=\frac{1}{4\pi^{2}}\int w_{cl}\left(X|\mu ,\nu \right)\exp\left(i\left(X-\mu q-\nu p\right)\right)dXd\mu d\nu .$$

One can check that the function $w_{cl}\left(X|\mu ,\nu \right)$ is non-negative and normalized

(7) $$\int w_{cl}\left(X|\mu ,\nu \right)dX=1.$$

The position X=μq+νp is one random variable and the real parameters μ and ν determine the reference frames in the particle phase-space where the position is measured. Thus, the relations (5) and (6) provide the invertible map of the probability density f(q,p) onto the conditional probability density of one random position *X*, measured in a reference frame with known parameters μ=scosθ, $\nu =s^{-1}\sin\theta$. These parameters mean that, if the initial reference axes provide axes *q* and *p*, then we construct the rescaled axis $q^{\prime }=sq$, $p^{\prime }=s^{-1}p$ and then rotate these axes obtaining $q^{\prime \prime }=\cos\theta q^{\prime }+\sin\theta p^{\prime }$, $p^{\prime \prime }=-\sin\theta q^{\prime }+\cos\theta p^{\prime }$. Thus, the meaning of the variable *X* is as follows: It is the particle position measured in all the rescaled and rotated reference frames. The Radon map provides the possibility for a classical particle to introduce [22] the tomographic probability distribution of the state usually determined by the probability density f(q,p). The rescaling and rotation of the axis in phase-space of the particle is a linear symplectic transform and the function $w_{cl}\left(X|\mu ,\nu \right)$ is called the symplectic tomogram of the classical particle state. Since the map is invertible, the tomogram contains the same information about the state that is contained in the usual probability distribution f(q,p). It turns out that the quantum version of the map provides the possibility to construct the invertible map for the particle state in quantum mechanics by relating the density operator $\hat{\rho }$ of the state with the symplectic tomogram, using the following quantum version of the map, namely, introducing the symplectic tomogram, as follows:

(8) $$w_{\hat{\rho }}\left(X|\mu ,\nu \right)=\mathrm{Tr}\hat{\rho }\delta \left(X\hat{1}-\mu \hat{q}-\nu \hat{p}\right)$$

and introducing the dequantizer operator $\delta (X\hat{1}-\mu \hat{q}-\nu \hat{p})$. All the known quasidistribution functions are obtained using different pairs of operators—quantizer operator $\hat{D}\left(x\right)$ and dequantizer operator $\hat{U}\left(x\right)$, where $x=x_{1},x_{2},\ldots x_{n}$.

These operators create the possibility of mapping the operators $\hat{A}$ acting in the Hilbert space, where the position $\hat{q}$ and the momentum $\hat{p}$ act, by following the generic map of operators $\hat{A}\to f_{A}\left(x\right)$ given by the formula for the function $f_{A}\left(x\right)$ called the symbol of the operator $\hat{A}$

(9) $$f_{A}\left(x\right)=\mathrm{Tr}\left(\hat{A}\hat{U}\left(x\right)\right).$$

The inverse transform $f_{A}\left(x\right)\to \hat{A}$ is given by the formula

(10) $$\hat{A}=\int f_{A}\left(x\right)\hat{D}\left(x\right)dx,$$

where the quantizer operators $\hat{D}\left(x\right)$ provide the possibility of reconstructing the operator $\hat{A}$ if its symbol $f_{A}\left(x\right)$ is known. The map given by Equations (9) and (11) provides the possibility of introducing the star-product of functions $f_{A}\left(x\right)$ and $f_{B}\left(x\right)$, which are symbols of the operators $\hat{A}$ and $\hat{B}$. The symbol of the operator $\hat{A}\hat{B}$ is given by the formula

(11) $$f_{AB}\left(x\right)=\mathrm{Tr}\left(\hat{A}\hat{B}\hat{U}\left(x\right)\right).$$

Using the relations (9) and (11) the star-product of the function $f_{A}\left(x\right)$ and $f_{B}\left(x\right)$

(12) $$\left(f_{A}★f_{B}\right)\left(x\right)=f_{AB}\left(x\right)$$

is presented in the integral form

(13) $$\left(f_{A}★f_{B}\right)\left(x\right)=\int f_{A}\left(x_{1}\right)f_{B}\left(x_{2}\right)K\left(x_{1},x_{2},x\right)dx_{1}dx_{2},$$

with the kernel which is easy to express in terms of the quantizer–dequantizer

(14) $$K\left(x_{1},x_{2},x\right)=\mathrm{Tr}\left(\hat{D}\left(x_{1}\right)\hat{D}\left(x_{2}\right)\hat{U}\left(x\right)\right).$$

Since the product of operators is associative, i.e., $\left(\left(\hat{A}\hat{B}\right)\hat{C}\right)=\left(\hat{A}\left(\hat{B}\hat{C}\right)\right)$, the star-product of the symbols of the operators is also associative. For the symplectic tomogram, the inverse quantum Radon transform reads [23]

(15) $$\hat{\rho }=\frac{1}{2\pi }\int w\left(X|\mu ,\nu \right)\exp\left(i(X\hat{1}-\mu \hat{q}-\nu \hat{p})\right)dXd\mu d\nu .$$

This means that the quantizer operator for the symplectic tomography method has the form

(16) $$\hat{D}\left(X|\mu ,\nu \right)=\frac{1}{2\pi }\exp\left(i(X\hat{1}-\mu \hat{q}-\nu \hat{p})\right).$$

Thus, we have $x=(x_{1},x_{2},x_{3})=X,\mu ,\nu$ and the dequantizer reads

(17) $$\hat{U}\left(X|\mu ,\nu \right)=\delta \left(i(X\hat{1}-\mu \hat{q}-\nu \hat{p})\right).$$

The kernel describing the star-product of the operators in symplectic tomography is expressed as follows:

(18) $$\begin{array}{ccc} & & \hat{K}\left(X_{1},\mu_{1},\nu_{1},X_{2},\mu_{2},\nu_{2},X,\mu ,\nu \right)=\frac{1}{4\pi^{2}}Tr\left[\exp\left(i(X_{1}\hat{1}-\mu_{1}\hat{q}-\nu_{1}\hat{p})\right)\right.\\ & & \times \left.\exp\left(i(X_{2}\hat{1}-\mu_{2}\hat{q}-\nu_{2}\hat{p})\right)\delta \left(i(X\hat{1}-\mu \hat{q}-\nu \hat{p})\right)\right]. \end{array}$$

In an explicit form, it reads

(19) $$\begin{array}{ccc} & & \hat{K}\left(X_{1},\mu_{1},\nu_{1},X_{2},\mu_{2},\nu_{2},X,\mu ,\nu \right)=\frac{1}{4\pi^{2}}\delta \left(\mu \left(\nu_{1}+\nu_{2}\right)-\nu \left(\mu_{1}+\mu_{2}\right)\right)\\ & & \times \exp\left(\frac{i}{2}\left(\nu_{1}\mu_{2}-\nu_{2}\mu_{1}+2X_{1}+2X_{2}-2\frac{\nu_{1}+\nu_{2}}{\nu }X\right)\right). \end{array}$$

One can use the formalism of quantizer–dequantizer operators to write the evolution equation for the symbols of the density operators. For example, the von Neumann equation for the density operator $\hat{\rho }\left(t\right)$ is written in the form (we use m=ω=ħ=1)

(20) $$\frac{\partial \hat{\rho }}{\partial t}+i\left(\hat{H}\left(t\right)\hat{\rho }\left(t\right)-\hat{\rho }\left(t\right)\hat{H}\left(t\right)\right)=0.$$

Here, $\hat{\rho }\left(t\right)$ has the symbol $f_{\rho }\left(x,t\right)$ and the Hamiltonian operator $\hat{H}\left(t\right)$ has the symbol $f_{H}\left(x,t\right)$, where we consider the symbols for arbitrary quasidistributions corresponding to quantizer–dequantizer operators. Then Equation (20) takes the form

(21) $$\frac{\partial f_{\rho }\left(x,t\right)}{\partial t}+i\left(f_{H}★f_{\rho }-f_{\rho }★f_{H}\right)\left(x,t\right)=0.$$

The equation for the given Hamiltonian $\hat{H}\left(t\right)$ has the general form of an integral equation

(22) $$\frac{\partial f_{\rho }\left(x,t\right)}{\partial t}+i\int \left(f_{H}\left(x_{1},t\right)f_{\rho }\left(x_{2},t\right)-f_{\rho }\left(x_{1},t\right)f_{H}\left(x_{2},t\right)\right)K\left(x_{1},x_{2},x\right)dx_{1}dx_{2}=0.$$

Here, the symbol of the Hamiltonian $f_{H}\left(x_{1},t\right)=\mathrm{Tr}\left(\hat{H}\left(t\right)\hat{U}\left(x_{1}\right)\right)$ and the symbol of the density operator $f_{\rho }\left(x_{2},t\right)=\mathrm{Tr}\left(\hat{\rho }\left(t\right)\hat{U}\left(x_{2}\right)\right).$ In the case of a harmonic oscillator in the tomographic probability representation, the symbol of the density operator $\hat{\rho }\left(t\right)$ is given by the probability distribution function, (x=X,μ,ν),

(23) $$w_{\rho }\left(X|\mu ,\nu ,t\right)=f_{\rho }\left(x,t\right)=\mathrm{Tr}\hat{\rho }\left(t\right)\delta \left(X\hat{1}-\mu \hat{q}-\nu \hat{p}\right),\;\;f_{H}\left(x,t\right)=\mathrm{Tr}\hat{H}\left(t\right)\delta \left(X\hat{1}-\mu \hat{q}-\nu \hat{p}\right)$$

and the kernel of the associative product is the function (19). The Equation (22) is the kinetic equation for a probability distribution function

(24) $$\frac{\partial f_{\rho }\left(x,t\right)}{\partial t}+i\int f_{H}\left(x_{1},t\right)f_{\rho }\left(x_{2},t\right)\left[K\left(x_{1},x_{2},x\right)-K\left(x_{2},x_{1},x\right)\right]dx_{1}dx_{2}=0.$$

For symplectic tomography, the integral linear equation has the form

(25) $$\frac{\partial w_{\rho }\left(X|\mu ,\nu ,t\right)}{\partial t}+i\int w_{\rho }\left(X_{2}|\mu_{2},\nu_{2},t\right)K\left(X,\mu ,\nu ,X_{2},\mu_{2},\nu_{2},t\right)dX_{2}d\mu_{2}d\nu_{2}=0.$$

Here,

(26) $$\begin{array}{ccc} & & K(X,\mu ,\nu ,X_{2},\mu_{2},\nu_{2},t)=\\ & & \int \left[K\left(X_{1},\mu_{1},\nu_{1},X_{2},\mu_{2},\nu_{2},t\right)-K\left(X_{2},\mu_{2},\nu_{2},X_{1},\mu_{1},\nu_{1},t\right)\right]f_{H}\left(X_{1},\mu_{1},\nu_{1},t\right)dX_{1}d\mu_{1}d\nu_{1}. \end{array}$$

## 3. Inverted Oscillator

Let us study the properties of the classical and quantum-inverted oscillator. The Hamiltonian of the classical inverted oscillator reads (m=ω=ħ=1)

(27) $$H_{cl}\left(q,p\right)=\frac{p^{2}}{2}-\frac{q^{2}}{2}.$$

One can check that the two expressions $q_{0}\left(q,p,t\right)$ and $p_{0}\left(q,p,t\right)$, satisfying the initial conditions $q_{0}\left(q,p,t=0\right)=q$ and $p_{0}\left(q,p,t=0\right)=p$ of the form

(28) $$q_{o}\left(q,p,t\right)=q\cosh t-p\sinh t,$$

(29) $$p_{o}\left(q,p,t\right)=-q\sinh t+p\cosh t,$$

satisfy the differential equations for any integral of motion I(q,p,t) for an inverted oscillator

(30) $$\frac{dI(q,p,t)}{dt}=\frac{\partial I(q,p,t)}{\partial t}+\frac{\partial I(q,p,t)}{\partial q}p-\frac{\partial I(q,p,t)}{\partial p}q=0.$$

One can check that, for a quantum-inverted oscillator with the Hamiltonian

(31) $$\hat{H}=\frac{{\hat{p}}^{2}}{2}-\frac{{\hat{q}}^{2}}{2}$$

the non-Hermitian operators

(32) $${\hat{q}}_{o}\left(\hat{q},\hat{p},t\right)=\hat{q}\cosh t-\hat{p}\sinh t,$$

(33) $${\hat{p}}_{o}\left(\hat{q},\hat{p},t\right)=-\hat{q}\sinh t+\hat{p}\cosh t$$

satisfy the equations for quantum integrals of motion $\hat{I}\left(\hat{q},\hat{p},t\right)$ of the form

(34) $$\frac{d\hat{I}\left(\hat{q},\hat{p},t\right)}{dt}=\frac{\partial \hat{I}\left(\hat{q},\hat{p},t\right)}{\partial t}+i\left[\hat{H},\hat{I}\left(\hat{q},\hat{p},t\right)\right]=0.$$

Also,

(35) $${\hat{q}}_{o}\left(\hat{q},\hat{p},t=0\right)=\hat{q}\;\mathrm{and}\;{\hat{p}}_{0}\left(\hat{q},\hat{p},t=0\right)=\hat{p}.$$

The Green function of the Schrödinger equation in position representation G(y,x,t) satisfies the system of equations [24]

(36) $$\left(y\cosh t+i\sinh t\frac{\partial }{\partial y}\right)G\left(y,x,t\right)=xG\left(y,x,t\right),$$

(37) $$\left(-i\cosh t\frac{\partial }{\partial y}-y\sinh t\right)G\left(y,x,t\right)=\left(i\frac{\partial }{\partial x}\right)G\left(y,x,t\right),$$

also

$$i\frac{\partial }{\partial t}G\left(y,x,t\right)=\left(-\frac{1}{2}\frac{\partial^{2}}{\partial y^{2}}-\frac{y^{2}}{2}\right)G\left(y,x,t\right),$$

and the initial condition for the Green function, i.e., for t=0 reads G(y,x,0)=δ(y−x). The Green function is analogous to the Green function of the usual harmonic oscillator with frequency $\omega^{2}=1$, but the condition $\omega^{2}=-1$ is used to obtain the expression for the propagator of the inverted oscillator from the propagator of the usual harmonic oscillator. We obtain

(38) $$G\left(y,x,t\right)=\frac{1}{\sqrt{2i\pi \sinh t}}\exp\left[\frac{i}{2}\left(\coth t\left(y^{2}+x^{2}\right)-\frac{2xy}{\sinh t}\right)\right].$$

Using the Green function and the initial condition for the wave function of the inverted oscillator, we can calculate the evolution of the wave function. This wave function in the moment t>0 determines the tomographic probability distribution describing the inverted oscillator. In fact, this integral relation of the wave function with the tomogram for the system with linear position and momentum integrals of motion provides the possibility of obtaining the evolution of the tomographic probability distribution of the inverted oscillator using a simple tool related to the Heisenberg position and momentum operators which will be shown later. Moreover, one can use other integrals of motion, which are constructed below.

The operators (integrals of motion)

(39) $$\hat{a}\left(t\right)=\frac{1}{\sqrt{2}}\left({\hat{q}}_{0}\left(t\right)+i{\hat{p}}_{0}\left(t\right)\right),$$

(40) $${\hat{a}}^{†}\left(t\right)=\frac{1}{\sqrt{2}}\left({\hat{q}}_{0}\left(t\right)-i{\hat{p}}_{0}\left(t\right)\right)$$

satisfy the commutation relations

(41) $$\left[\hat{a}\left(t\right),{\hat{a}}^{†}\left(t\right)\right]=\hat{1}$$

and

(42) $$\hat{a}\left(0\right)=\frac{1}{\sqrt{2}}\left(\hat{q}+i\hat{p}\right),\;{\hat{a}}^{†}\left(0\right)=\frac{1}{\sqrt{2}}\left(\hat{q}-i\hat{p}\right)$$

are standard annihilation and creation bosonic operators. There are other operators which are integrals of motion for the inverted oscillator [14] and the Hermitian one

(43) $$\hat{A}\left(t\right)=\frac{1}{\sqrt{2}}\left({\hat{q}}_{0}\left(t\right)+{\hat{p}}_{0}\left(t\right)\right),$$

(44) $${\hat{A}}^{†}\left(t\right)=\frac{1}{\sqrt{2}}\left({\hat{q}}_{0}\left(t\right)-{\hat{p}}_{0}\left(t\right)\right).$$

They satisfy the commutation relations

(45) $$\left[\hat{A}\left(t\right),{\hat{A}}^{†}\left(t\right)\right]=-i\hat{1}.$$

We construct the coherent states of the inverted oscillator |α,t〉 using the definition $\hat{a}\left(t\right)|\alpha ,t\rangle =\alpha |\alpha ,t\rangle .$ For t=0, this state reads

(46) $$\psi_{\alpha }\left(x,0\right)=\frac{1}{\pi^{1/4}}\exp\left(-\frac{{\left|\alpha \right|}^{2}}{2}-\frac{x^{2}}{2}+\sqrt{2}\alpha x-\frac{\alpha^{2}}{2}\right).$$

In addition to consideration of the inverted oscillator tomogram, we will address the evolution of the tomogram of the parametric oscillator with time-dependent frequency $\omega^{2}\left(t\right)$ with the Hamiltonian $\hat{H}=\frac{{\hat{p}}^{2}}{2}+\frac{\omega^{2}\left(t\right){\hat{q}}^{2}}{2}$, $\omega^{2}\left(0\right)=1$. The wave function of the states of the parametric oscillator was discussed in [24]. The parametric oscillator state, which is an analog of the ground state of the usual oscillator, satisfies the condition $\hat{a}\left(t\right)\psi_{0}\left(x,t\right)=0$. The normalized wave function is of the form

(47) $$\psi_{0}\left(x,t\right)=\frac{1}{\sqrt{\pi^{1/2}\epsilon \left(t\right)}}\exp\left(\frac{i\dot{\epsilon }\left(t\right)}{2\epsilon (t)}x^{2}\right).$$

The complex function ϵ(t) satisfies the equation

$$\ddot{\epsilon }\left(t\right)+\omega^{2}\left(t\right)\epsilon \left(t\right)=0.$$

The initial conditions for the function ϵ(t) are

$$\epsilon \left(0\right)=1,\;\dot{\epsilon }\left(0\right)=i.$$

According to the relation of the wave function with the tomogram of the state, which we mentioned above for the inverted oscillator state, for the state of the parametric oscillator with the wave Function (47), the tomogram in integral form reads

$$w_{0}\left(X|\mu ,\nu \right)=\frac{1}{2\pi |\nu |}{\left|\int \frac{1}{\sqrt{\pi^{1/2}\epsilon \left(t\right)}}\exp\left(\frac{i\dot{\epsilon }\left(t\right)}{2\epsilon (t)}y^{2}+\frac{i\mu y^{2}}{2\nu }-\frac{iXy}{\nu }\right)dy\right|}^{2}.$$

This is a Gaussian integral and we get the standard normal probability distribution with a zero mean value random position and time-dependent dispersions. For the usual time-independent frequency $\omega^{2}=1$ function $\epsilon \left(t\right)=e^{it}$. We have a normal probability distribution describing the quantum state of the parametric harmonic oscillator. One can see that the Hamiltonian of the inverted osciillator (27) has symmetry related to the group SO(1,1) and the usual oscillator Hamiltonian has symmetry related to the group SO(2). This means that the states of both oscillators can be related to irreducible representations of these groups. In principle, this means that the tomographic probability distributions related to the states of the inverted oscillator can be associated with the symmetry groups. We can try to solve the problem to find the relation of the irreducible representation of the Hamiltonian symmetry group with the tomographic probability distributions describing the Hilbert space state-vectors (or density operators) associated with the inverted oscillator states properties. This problem will be investigated in future publications following the approach considered for the hydrogen atom in the review [25], where the dynamical group O(4,2) was used as the symmetry of the Hamiltonian.

## 4. Tomograms of Evolving States of the Inverted Oscillator Prepared Initially in the Potential of the Usual Oscillator

Let us solve the following problem to describe the behavior of the symplectic tomograms of the inverted oscillator states, which, initially at t=0, were prepared as states of the usual oscillator with frequency ω=1 (and m=ħ=1). This means that the ground states of the usual oscillator with the Hamiltonian $\hat{H}={\hat{p}}^{2}/2+{\hat{q}}^{2}/2$, i.e., the oscillator with a wave function in position representation

(48) $$\psi_{0}\left(x,t=0\right)=\frac{1}{\pi^{1/4}}\exp\left(-\frac{x^{2}}{2}\right)$$

starts to evolve in potential $U\left(q\right)=-{\hat{q}}^{2}/2.$

The tomogram of the state with the initial wave function (48) $w_{0}\left(X|\mu ,\nu ,t=0\right)$ has the form given by the relation

(49) $$w_{0}\left(X|\mu ,\nu ,t=0\right)=\frac{1}{2\pi |\nu |}{\left|\int \frac{1}{\pi^{1/4}}\exp\left(-\frac{y^{2}}{2}+\frac{i\mu y^{2}}{2\nu }-\frac{iXy}{\nu }\right)dy\right|}^{2},$$

i.e.,

(50) $$w_{0}\left(X,\mu ,\nu ,t=0\right)=\frac{1}{\sqrt{\pi (\mu^{2}+\nu^{2})}}\exp\left(-\frac{X^{2}}{\mu^{2}+\nu^{2}}\right).$$

The evolution of this tomogram obeys (25) and (26). One can solve this integral equation, but we demonstrate the possibility to obtain the solution using the analogous relation (8)

(51) $$w_{o}\left(X|\mu ,\nu ,t\right)=\mathrm{Tr}\hat{\rho }\left(t\right)\delta \left(X\hat{1}-\mu \hat{q}-\nu \hat{p}\right),$$

where $\hat{\rho }\left(t\right)=e^{-i\hat{H}t}\hat{\rho }\left(0\right)e^{i\hat{H}t}$ and where $\hat{H}$ is given by (31). The relation (51) can be used in the form

(52) $$w_{o}\left(X|\mu ,\nu ,t\right)=\mathrm{Tr}\hat{\rho }\left(0\right)\delta \left(X\hat{1}-\mu e^{-i\hat{H}t}\hat{q}e^{i\hat{H}t}-\nu e^{-i\hat{H}t}\hat{p}e^{i\hat{H}t}\right)$$

Here, we used the property of the calculating trace of the operator product: $\mathrm{Tr}\left(\hat{A}\hat{B}\hat{C}\right)=\mathrm{Tr}\left(\hat{B}\hat{C}\hat{A}\right)$ applied to any function $f(\hat{A}\hat{B}\hat{C})$, including the Dirac delta-function. But we can then use the properties of the position $\hat{q}$ and the momentum $\hat{p}$ operators and these operators in the Heisenberg representation ${\hat{q}}_{H}\left(t\right)$ and ${\hat{p}}_{H}\left(t\right)$, which, for the inverted oscillator, have a form formally repeating the classical formula. This creates the possibility of solving the kinetic Equations (25) and (26) by replacement in the known symplectic tomographic probability representation formula of the parameters μ and ν by parameters $\bar{\mu }$, $\bar{\nu }$, expressing the relations of the ${\hat{q}}_{H}\left(t\right)$ and ${\hat{p}}_{H}\left(t\right)$, with the operators $\hat{q}$ and $\hat{p}$. This is easy to do for systems with a quadratic in the position $\hat{q}$ and momentum $\hat{p}$ operators, where we consider the equality

(53) $${\bar{\mu }}_{H}\hat{q}+{\bar{\nu }}_{H}\hat{p}=\mu {\hat{q}}_{H}\left(t\right)+\nu {\hat{p}}_{H}\left(t\right).$$

For this, we rewrite this equality in the form

(54) $$\mu \left(\cosh t\hat{q}+\sinh t\hat{p}\right)+\nu \left(\sinh t\hat{q}+\cosh t\hat{p}\right)=\mu_{H}\hat{q}+\nu_{H}\hat{p}.$$

This means that

(55) $$\mu_{H}=\mu \cosh t+\mu \sinh t\;\mathrm{and}\;\nu_{H}=\mu \sinh t+\mu \cosh t.$$

Then, we can write the symplectic tomogram as the solution of Equations (25) and (26) for the state of the inverted oscillator, with the additional condition that its initial value at time *t* was given by (25) and (26)

(56) $$\begin{array}{ccc} & & w_{0}\left(X|\mu ,\nu ,t\right)=\frac{1}{\sqrt{\pi \left(\left(\mu^{2}+\nu^{2}\right)\left(2\sinh^{2}t+1\right)+2\mu \nu \sinh2t\right)}}\\ & & \exp\left(-\frac{X^{2}}{\left(\mu^{2}+\nu^{2}\right)\left(2\sinh^{2}t+1\right)+2\mu \nu \sinh2t}\right). \end{array}$$

This is the normal probability distribution with zero mean value and varying dispersions. The physical meaning of the tomogram means that, for μ=1, ν=0, the relation (56) provides the probability distribution of the position; for μ=0,ν=1, it is equal to the probability distribution of the momentum of the inverted oscillator at time *t* prepared in the initial time in the ground state of the usual oscillator. The dispersions of the position and momentum are equal and satisfy the Heisenberg uncertainty relation at any time *t*. The correlation coefficient *r* is given by the equality in the Robertson–Schrödinger uncertainty relation

(57) $${\left(\delta q\right)}^{2}{\left(\delta p\right)}^{2}\geq \frac{1}{4(1-r^{2})};\;{\left(\delta q\right)}^{2}={\left(\delta p\right)}^{2}=\frac{1}{2}+\sinh^{2}t.$$

This means that the correlation of the position and the momentum coefficient 1≥r≥0 satisfies the condition

(58) $$r^{2}=1-\frac{1}{4{\left(\frac{1}{2}+\sinh^{2}t\right)}^{2}}.$$

For t=0, the correlation is equal to zero and for large time periods it approaches to unity. This means that large fluctuations are present with large kinetic energy contributions to the behaviour of the inverted oscillator. Following our approach, we consider the behavior of the inverted oscillator for a system with time-dependent frequency, such that the frequency $\omega^{2}\left(t\right)$ depends on time and $\omega^{2}\left(t\right)$ for t<0 equals one and for t≥0, $\omega^{2}=-1$. We consider such a system if the initial state of the oscillator is a coherent state with wave function $\psi_{\alpha }\left(x,t\right)$. The tomographic probability distribution in this case is a normal probability distribution. Using the approach $\mu \to \bar{\mu }$, $\nu \to \bar{\nu }$, we get the time evolution of the inverted oscillator tomogram of the form

(59) w0(X|μ,ν,t)=1π(μ2+ν2)(2sinh2t+1)+2μνsinh2texp−X−2(μcosht+νsinht)Reα−2(νcosht+μsinht)Imα2(μ2+ν2)(2sinh2t+1)+2μνsinh2t.

For the initial state with the wave function of the Fock state and its tomogram for t>0, we have the evolving symplectic tomogram $w_{n}\left(X|\mu ,\nu ,t\right)$ with replacement $\mu \to \mu_{H}\left(t\right),$$\nu \to \nu_{H}\left(t\right)$, i.e.,

(60) $$w_{n}\left(X|\mu ,\nu ,t\right)=\frac{1}{2^{n}n!}w_{0}\left(X|\mu ,\nu ,t\right){\left|H_{n}\left(\frac{X}{\left(\mu^{2}+\nu^{2}\right)\left(2\sinh^{2}t+1\right)+2\mu \nu \sinh2t}\right)\right|}^{2}.$$

One can introduce the entropy of the quantum states of the inverted oscillator with the tomogram w(X|μ,ν,t) using the Schannon entropy

(61) S(μ,ν,t)=−∫w(X|μ,ν,t)lnw(X|μ,ν,t)dX.

For the Gaussian states with wave initial functions (46), the initial entropy S(μ,ν,t=0) reads

(62) $$S\left(\mu ,\nu ,t=0\right)=\sqrt{\pi e(\mu^{2}+\nu^{2})}.$$

For the inverted oscillator, the entropy at time *t* is expressed in terms of the initial value of the entropy S(μ,ν,t=0)

(63) $$S\left(\mu ,\nu ,t\right)=\sqrt{\pi e({\left(\mu \cosh t+\nu \sinh t\right)}^{2}+{\left(\nu \cosh t+\mu \sinh t\right)}^{2})}.$$

## 5. Tomograms of Inverted Oscillator States

Let us construct the tomograms of the coherent state of the inverted oscillator. The tomographic probability distribution of the coherent state of the oscillator reads

(64) $$w_{\alpha }\left(X|\mu ,\nu ,t=0\right)=\mathrm{Tr}\left[|\psi_{\alpha }\rangle \langle \psi_{\alpha }|\delta \left(X-\mu \hat{q}-\nu \hat{p}\right)\right].$$

The explicit expression, in integral form, of the coherent state with the wave Function (46) reads [26]

(65) $$w_{\alpha }\left(X|\mu ,\nu \right)=\frac{1}{2\pi |\nu |}{\left|\int \psi_{\alpha }\left(y\right)\exp\left(\frac{i\mu y^{2}}{2\nu }-\frac{iXy}{\nu }\right)dy\right|}^{2}.$$

Thus, we have normal probability distributions

(66) $$w_{\alpha }\left(X|\mu ,\nu \right)=\frac{1}{\sqrt{\pi (\mu^{2}+\nu^{2})}}\exp\left[-\frac{{\left(X-{\bar{X}}_{\alpha }\right)}^{2}}{\mu^{2}+\nu^{2}}\right],$$

where X¯=μ2Reα+ν2Imα. The function (46) is the eigenfunction of the integral of motion (43) taken for t=0, i. e.,

(67) $$\frac{1}{\sqrt{2}}\left(\hat{q}+i\hat{p}\right)\psi_{\alpha }\left(x,0\right)=\alpha \psi_{\alpha }\left(x,0\right).$$

If we write the tomogram of the initial coherent state of the inverted oscillator, we have to calculate the following trace:

(68) $$w_{\alpha }\left(X|\mu ,\nu ,t\right)=\mathrm{Tr}{\hat{\rho }}_{\alpha }\left(t\right)\delta \left(X\hat{1}-\mu \hat{q}-\nu \hat{p}\right).$$

Since ${\hat{\rho }}_{\alpha }\left(t\right)=\hat{U}\left(t\right){\hat{\rho }}_{\alpha }\left(0\right){\hat{U}}^{†}\left(t\right)$, where $\hat{U}\left(t\right)=\exp\left(-i\hat{H}t\right)$ and $\hat{H}=\frac{{\hat{p}}^{2}}{2}-\frac{{\hat{q}}^{2}}{2}$, we get

(69) $$w_{\alpha }\left(X|\mu ,\nu ,t\right)=\mathrm{Tr}\hat{\rho }\left(0\right)\delta \left(X\hat{1}-\mu {\hat{U}}^{†}\left(t\right)\hat{q}\hat{U}\left(t\right)-\nu {\hat{U}}^{†}\hat{p}\hat{U}\left(t\right)\right).$$

The operators ${\hat{U}}^{†}\left(t\right)\hat{q}\hat{U}\left(t\right)$ and ${\hat{U}}^{†}\left(t\right)\hat{p}\hat{U}\left(t\right)$ are the Heisenberg operators of position and momentum ${\hat{q}}_{H}\left(t\right)$ and ${\hat{p}}_{H}\left(t\right)$. Using the integrals of motion (43) and (44) at time −t, we get these operators

(70) $${\hat{q}}_{H}\left(t\right)=\hat{q}\cosh t+\hat{p}\sinh t,$$

(71) $${\hat{p}}_{H}\left(t\right)=\hat{q}\sinh t+\hat{p}\cosh t.$$

This means that the arguments of the delta-function take the form $X\hat{1}-\mu {\hat{q}}_{H}-\nu {\hat{p}}_{H}=\bar{X}\hat{1}-\mu_{H}\hat{q}-\nu_{H}\hat{p}$. Here $\bar{X}=X$, $\mu_{H}=\mu \sinh t+\nu \cosh t$. This means that the function (68) can be calculated and the result gives the coherent states of the inverted oscillator at time *t* with the initial state (66)

(72) $$\begin{array}{ccc} & & w_{\alpha }\left(X|\mu ,\nu ,t\right)=\frac{1}{\sqrt{\pi \left[{\left(\mu \sinh t+\nu \cosh t\right)}^{2}+{\left(\nu \sinh t+\mu \cosh t\right)}^{2}\right]}}\\ & & \exp\left[-\frac{{\left(X-\bar{X}\right)}^{2}}{{\left(\mu \sinh t+\nu \cosh t\right)}^{2}+{\left(\nu \sinh t+\mu \cosh t\right)}^{2}}\right], \end{array}$$

where

(73) X¯=2Reα(μsinht+νcosht)+Imα(μcosht+νsinht).

Analogously, we can calculate the states with wave function $\psi_{n}\left(x\right)$ of the inverted oscillator for which ${\hat{a}}^{†}\hat{a}\psi_{n}\left(x\right)=n\psi_{n}\left(x\right)$, and which has the form

(74) $$\psi_{n}\left(x\right)=\frac{e^{-x^{2}/2}}{\pi^{1/4}}\frac{1}{\sqrt{2^{n}n!}}H_{n}\left(x\right),$$

where $H_{n}\left(x\right)$ is the Hermite polynomial. Then the tomogram of this state reads

(75) $$w_{n}\left(X|\mu ,\nu \right)=\frac{1}{2^{n}n!}\exp\left(-\frac{X^{2}}{\mu^{2}+\nu^{2}}\right){\left|H_{n}\left(\frac{X}{\mu^{2}+\nu^{2}}\right)\right|}^{2}\frac{1}{\sqrt{\pi (\mu^{2}+\nu^{2})}}.$$

The tomogram of the eigenstates $\psi_{n}\left(x,t\right)$ of the operators ${\hat{a}}^{†}\left(t\right)\hat{a}\left(t\right)$, i.e., ${\hat{a}}^{†}\left(t\right)\hat{a}\left(t\right)\psi_{n}\left(x,t\right)=n\psi_{n}\left(x,t\right)$, n=0,1,2,… can be calculated and the result is given by Equation (75) with replacement $\mu =\bar{\mu }$ and $\nu =\bar{\nu }$. Thus, we have the tomogram of the Fock state of the inverted oscillator, i.e.,

(76) $$\begin{array}{ccc} & & w_{n}\left(X,\mu ,\nu ,t\right)=\frac{1}{2^{n}n!}\exp\left[-\frac{X^{2}}{{\left(\mu \sinh t+\nu \cosh t\right)}^{2}+{\left(\nu \sinh t+\mu \cosh t\right)}^{2}}\right]\\ & & \times {\left|H_{n}\left(\frac{X}{\sqrt{{\left(\mu \sinh t+\nu \cosh t\right)}^{2}+{\left(\nu \sinh t+\mu \cosh t\right)}^{2}}}\right)\right|}^{2}\\ & & \times \frac{1}{\sqrt{\pi \left[{\left(\mu \sinh t+\nu \cosh t\right)}^{2}+{\left(\nu \sinh t+\mu \cosh t\right)}^{2}\right]}}, \end{array}$$

where X¯=2μcosht+νsinhtReα+μsinht+νcoshtImα.

## 6. Conclusions

To conclude, we summarize the main results of the work and provide some general observations. We studied the evolution of coherent and Fock states prepared using the potential of a conventional oscillator, but moved later to the potential of an inverted oscillator or parametric oscillator. The description of the states of the inverted oscillator was used in the probability representation of quantum mechanics, where the state of the inverted oscillator was identified with a probability distribution function. The same property of the evolving state of the parametric oscillator was found. For construction of this probability distribution, we used the formalism of quantizer–dequantizer operators and applied this formalism to the new integral equation for the probability distribution with an explicitly constructed integral kernel. Such equations can be extended and applied for all other known representations of quantum states, where the density operators of the states are mapped onto the functions–symbols of density operators, for which the star-product corresponding to the product of the operators is introduced. The evolution of the inverted oscillator coherent states, which is the evolution of the normal probability distributions, can also be generalized to other states, such as squeezed states and thermal oscillator states, as well as other representations of quantum states, such as groupoids representations [27]. The thermal oscillator states for negative temperature, as well as the structure of the Hilbert spaces of the inverted oscillator states using negative and positive Hilbert spaces [17], will be addressed in the tomographic probability representation. We can also extend application of the probability representation of quantum states to the problems of quantum-like systems discussed in [28,29,30,31]. We will investigate these problems in future papers.

## Data Availability

Not applicable.
